# HBX Multi‐Mutations Combined With Traditional Screening Indicators to Establish a Nomogram Contributes to Precisely Stratify the High‐Risk Population of Hepatocellular Carcinoma

**DOI:** 10.1002/cam4.70748

**Published:** 2025-03-05

**Authors:** Chao‐Jun Zhang, Xiao‐Mei Chen, Chang Yan, Rui‐Bo Lv, Sanchun An, Yun‐Xin Gao, Tian‐Ren Huang, Wei Deng

**Affiliations:** ^1^ Department of Experimental Research Guangxi Medical University Cancer Hospital Nanning Guangxi China; ^2^ Department of Radiation Oncology Guangxi Medical University Cancer Hospital Nanning Guangxi People's Republic of China; ^3^ Guangdong Forevergen Medical Technology Co Ltd Foshan Guangdong China; ^4^ Guangxi Cancer Molecular Medicine Engineering Research Center Nanning Guangxi China

**Keywords:** diagnostic, HBV mutation, hepatocellular carcinoma, nomogram, predictive model

## Abstract

**Background:**

Hepatocellular carcinoma (HCC) is one of the most prevalent malignant tumors, often diagnosed at an advanced stage with limited treatment options and a poor prognosis. The present study aimed to identify the risk factors (RFs) for HCC and develop a nomogram incorporating dominant HBX mutations to predict the risk of HCC occurrence in high‐risk (HR) populations.

**Methods:**

We collected early HCC screening and monitoring factors from cohorts of HCC patients and HR populations, including gender, age, AFP, ALT, as well as hepatitis B virus (HBV) infection and mutation indicators such as hepatitis B surface antigen (HBsAg), HBV DNA replication level, HBV genotype, and high‐frequency mutations in HBX. Independent predictive factors for HCC onset were determined through both univariate and multivariate logistic regression analyses. Two nomograms with and without HBX mutation data were established to predict the risk of HCC incidence in HR populations, and their performance was evaluated using calibration curves, receiver operating characteristic (ROC) curves, as well as decision curve analysis (DCA).

**Results:**

A total of 312 participants were included. Independent RFs for HCC onset were identified as A1762T+G1764A multi‐mutations, T1753C/G/A+A1762T+G1764A multi‐mutations, and ALT > 40 U/L. The area under the curve (AUC) of the diagnostic nomogram with HBX mutation data was 0.835 in the training set and 0.869 in the testing set for the nomogram. Besides, the AUC of the diagnostic nomogram without HBX mutation data in the training set was 0.798 and 0.818 in the testing set. The calibration curve together with DCA indicated that the nomogram containing HBX mutation data had better predictive performance.

**Conclusions:**

The established nomograms predicted the risk of HCC occurrence in HR populations with good accuracy, providing a valuable reference for precise stratification of HR populations and HCC screening.

## 1 Background

Hepatocellular carcinoma (HCC) ranked as the fourth most common cancer and the second leading cause of cancer‐related mortality in China in 2022 [1], posing a significant threat to human health. The majority of HCC patients are diagnosed at an advanced stage, resulting in limited treatment options and a 5‐year survival rate of less than 10% [2]. However, the 5‐year overall survival rate for early‐detected liver cancer can be as high as 69.0%–86.2% after comprehensive treatment [3]. Therefore, early detection and treatment through screening are effective prevention strategies to reduce the mortality rate of HCC and improve long‐term survival.

Since approximately 90% of HCC patients in China are infected with Hepatitis B virus (HBV), screening for HCC in HBV‐infected individuals offers greater health economic benefits in contrast with screening the general population. Current guidelines and consensus for the prevention and control of HCC in China recommend a screening program for serum alpha‐fetoprotein (AFP) along with liver ultrasonography every 6 months in hepatitis B surface antigen (HBsAg)‐positive populations. However, the high prevalence of HBV infections presents a significant challenge to this HCC screening program. The latest survey data showed that the prevalence of HBsAg in the general population was 5.8%, with 79 million cases of chronic HBV infection [4], accounting for about one‐third of the global infected population. Therefore, in addition to HBsAg, more non‐invasive HBV‐related markers for precise stratification of HR patients for HCC are needed for their early diagnosis and treatment.

Identifying and collecting risk prediction factors for HR populations to establish prediction models is an effective measure to improve early diagnosis rates of HCC. Researchers have established several risk prediction models for HBV‐related HCC to assess the occurrence and prognosis of HCC. For HBV‐infected individuals, there are the Risk Estimation for Chronic Hepatitis B (REACH‐B) model [5], Platelet Count, Age, Gender, and Hepatitis B e Antigen (PAGE‐B) model [6] and Age, Gender, and Elevated HBV DNA (AGED) model [7], with predictors including gender, age, serum ALT concentration, hepatitis B e antigen (HBeAg) status, serum HBV DNA level, and platelet count. However, due to technological limitations, HBV genetic variations have received little attention at the genetic level. In our previous study [8], we demonstrated the advantages of droplet digital PCR (ddPCR) for multi‐variation detection in early HCC screening compared to Next‐Generation Sequencing (NGS). We also confirmed that the combined mutations of G1512A+A1630G, A1762T+G1764A, as well as T1753C/G/A+A1762T+G1764A were more frequently expressed in early HCC cases. However, practical issues remain, including whether ddPCR technology is superior to the widely used quantitative polymerase chain reaction (qPCR); whether the inclusion of HBX multi‐mutation testing can improve the efficiency of the traditionally recognized HCC screening program; and how to enhance the accessibility of new screening technologies and implement them in large sample populations and primary medical settings. These issues need further validation.

In this study, we attempted to innovate by combining HBV genetic variation indicators (HBV genotype, high‐frequency HBX mutations, and multi‐mutations that refers to the presence of multiple mutation sites within a single sample, which can be simultaneously detected in a single test using ddPCR or other methods) and HBV infection indicators (HBsAg and HBV DNA level) with the traditional HBV‐related HCC screening program to determine whether the inclusion of HBX mutation data could enhance the predictive accuracy of HCC risk models. These indicators were assigned values as observed factors, with HCC occurrence as the outcome event. Univariate and multivariate logistic regression analyses were adopted to identify independent risk factors (RFs). A nomogram prediction model was then constructed to predict the risk of HCC occurrence in HR populations, aiming to evaluate the predictive ability of these novel indicators and corresponding models for HCC and provide a basis for precise stratification of HR populations and HCC screening.

## 2 Patients and Methods

### 2.1 Study Population Selection

This study included a total of 312 participants from a cohort established for early diagnosis and treatment screening in HR areas for HCC in Guangxi, China, since 2010. The participants underwent hepatectomy at Guangxi Medical University Cancer Hospital between 2013 and 2014. They were categorized into two groups: the HCC group (155 cases) and the high‐risk HCC group (157 cases). HCC inclusion criteria were as follows: (1) Diagnosis of HCC based on criteria established by the Chinese Medical Association; (2) Positive for HBsAg or HBV DNA; (3) Pathologically confirmed HCC; (4) No prior chemotherapy or radiotherapy before surgery; (5) Complete clinical data and relevant laboratory test results. Exclusion criteria were: (1) Metastatic liver tumor; (2) HCC not confirmed by pathological examination; (3) Previous chemotherapy or radiotherapy for HCC or other tumors; (4) Rheumatic and immune system diseases; (5) Incomplete clinical data or test results. This study was approved by the Ethics Committee of Guangxi Medical University Cancer Hospital (NO. 2021‐KY‐045), and informed consent was obtained from all participants.

We collected blood from the 312 participants as research samples. Whole blood samples were collected from all patients using standard blood collection tubes. Peripheral circulating blood was allowed to clot at room temperature for 1 h or kept at 2°C–8°C overnight. Serum was then separated by centrifugation at 3000 rpm for 10 min at room temperature, and the supernatant (serum) was carefully collected and stored at −80°C (avoiding repeated freeze–thaw cycles). The serum was used for the extraction of HBV DNA, sequencing, ddPCR, and other experiments.

### 2.2 HCC Screening

All subjects participated in regular physical examinations based on the traditional HCC screening program, during which data including age, gender, HBsAg, AFP, alanine aminotransferase (ALT) levels, and liver ultrasonography results were collected. ALT, AFP, and HBsAg were assessed by enzyme‐linked immunosorbent assay (ELISA) using kits from Nanjing Jiancheng (Supporting Informations). Liver ultrasonography examinations were performed by medical imaging specialists, and the results were reviewed by senior physicians.

### 2.3 Assessment of HBV Infection Status

HBV infection status was assessed using indicators including HBV DNA levels and HBV genotypes. HBV DNA levels were quantified by qPCR using the Zhongda Daan Gene Diagnostic Kit for HBV quantification. HBV DNA was extracted from serum samples using the QIAamp UltraSens Virus Kit (53704). A portion of the HBV S gene was amplified and sequenced by Sanger sequencing. The sequencing data were input into the NCBI Viral Genotyping Tool for HBV DNA genotype identification.

### 2.4 HBX Variations Examined by ddPCR and qPCR

High‐frequency mutations and multi‐mutations in the HBX gene were detected using ddPCR and qPCR. The sensitivity and specificity of these two methods were compared. Plasmids containing mutations such as G1512A, A1630G, T1753C, T1753G, T1753A, A1762T, and G1764A, along with wild‐type HBX, were constructed at different concentrations. Plasmid sequences (NC_003977.1) are provided in Table S1, and the primer and probe sequences for ddPCR and qPCR are listed in Table S2. For ddPCR, reactions were performed using the MicroDrop‐100A droplet digital PCR system (FOREVENRGEN) to quantify the copy number of HBV variations. The ddPCR reaction mixture (20 μL) included 5 μL of ddPCR reaction premix, 1800 nmol/L of primers, 500 nmol/L of probes, 2 μL of plasmid template, and 8.9 μL of diethylpyrocarbonate (DEPC)‐treated water. For qPCR, the reaction mixture (20 μL) included 5 μL of reaction premix, 800 nmol/L of primers, 500 nmol/L of probes, 2 μL of TB Green PrimeEX Taq II, 2 μL of plasmid template, and 5.9 μL of DEPC‐treated water. Reaction conditions for both methods are shown in Table S3. The optimized ddPCR method was used to assess HBX DNA mutations in the serum of the 312 study subjects.

### 2.5 Nomogram Establishment

To explore RFs for HCC occurrence, we constructed two nomograms using two different datasets: one including traditional RFs along with HBX mutation data, and another excluding HBX mutation data. This approach allowed us to investigate whether incorporating high‐frequency HBX mutations would improve the predictive performance of the model. Subjects were randomly divided into training sets (70%) and testing sets (30%). The training sets were used to develop the nomogram, while the testing sets were used for validation.

### 2.6 Statistical Analysis

Statistical analyses were conducted using SPSS 25.0 and R software (version 4.3.1). Variables between the training and testing sets were compared using the chi‐square test. A significance level of *p* < 0.05 was considered statistically significant. Univariate logistic regression analysis was used to identify factors associated with the occurrence of HCC. Variables with *p* < 0.05 in univariate analysis were included in multivariate binary logistic regression to identify independent RFs for HCC. Based on these independent predictive factors [9], prediction and prognosis calibration plots were developed using the rms package in R software. Receiver operating characteristic (ROC) curves were generated for prediction plots [10]. The area under the curve (AUC) was used to assess the discriminative ability of the prediction plots. Calibration curves for the nomogram [11] and decision curve analysis (DCA) curves were established to evaluate the model's performance.

## 3 Results

### 3.1 Characteristics of the Study Population

A total of 312 subjects were included in the study, with the participants randomized into training (218 cases) and testing (94 cases) sets at a ratio of 7:3. The data collected from the study subjects were processed as shown in Table 1. The differences between the two data sets in terms of age, gender, HBV infection status, and HBV variations were not statistically significant (all *p* > 0.05).

**TABLE 1:** Basic information data.

Characteristics	HCC group	HCC high risk group	*p*
*n*	155	157	
Gender, *n* (%)			0.604
Male	127 (81.9%)	125 (79.6%)	
Female	28 (18.1%)	32 (20.4%)	
Age, median (IQR)	50 (44, 59)	53 (46, 59)	0.106
G1512A, *n* (%)			0.368
G1512A mutaiton	151 (97.4%)	150 (95.5%)	
No G1512A mutation	4 (2.6%)	7 (4.5%)	
A1630G, *n* (%)			0.407
A1630G mutaiton	117 (75.5%)	112 (71.3%)	
No A1630G mutation	38 (24.5%)	45 (28.7%)	
G1512A+A1630G, *n* (%)			0.003
G1512A+A1630G co‐mutaiton	89 (57.4%)	115 (73.2%)	
No G1512A+A1630G co‐mutaiton	66 (42.6%)	42 (26.8%)	
T1753C/G/A, *n* (%)			0.484
T1753C/G/A mutaiton	155 (100%)	155 (98.7%)	
No T1753C/G/A mutation	0 (0%)	2 (1.3%)	
A1762T, *n* (%)			0.624
A1762T mutaiton	154 (99.4%)	154 (98.1%)	
No A1762T mutation	1 (0.6%)	3 (1.9%)	
G1764A, *n* (%)			0.004
G1764A mutaiton	129 (83.2%)	109 (69.4%)	
No G1764A mutation	26 (16.8%)	48 (30.6%)	
A1762T+G1764A, *n* (%)			0.001
A1762T+G1764A co‐mutaiton	52 (33.5%)	28 (17.8%)	
No A1762T+G1764A co‐mutaiton	103 (66.5%)	129 (82.2%)	
T1753C/G/A+A1762T, *n* (%)			0.497
T1753C/G/A+A1762T co‐mutaiton	154 (99.4%)	157 (100%)	
No T1753C/G/A+A1762T co‐mutaiton	1 (0.6%)	0 (0%)	
T1753C/G/A+G1764A, *n* (%)			0.626
T1753C/G/A+G1764A co‐mutaiton	148 (95.5%)	148 (94.3%)	
No T1753C/G/A+G1764A co‐mutaiton	7 (4.5%)	9 (5.7%)	
T1753C/G/A+A1762T+G1764A, *n* (%)			0.018
T1753C/G/A+A1762T+G1764A co‐mutaiton	94 (60.6%)	115 (73.2%)	
No T1753C/G/A+A1762T+G1764A co‐mutaiton	61 (39.4%)	42 (26.8%)	
HBV DNA copy number, *n* (%)			< 0.001
≥ 1 × 10^3^	122 (78.7%)	88 (56.1%)	
< 1 × 10^3^	33 (21.3%)	69 (44.9%)	
HbsAg, *n* (%)			< 0.001
Positive	144 (92.9%)	123 (78.3%)	
Negative	11 (7.1%)	34 (21.7%)	
AFP, *n* (%)			< 0.001
> 400 U/L	74 (47.7%)	157 (100%)	
≤ 400 U/L	81 (52.3%)	0 (0%)	
ALT, *n* (%)			< 0.001
> 40 U/L	117 (75.5%)	23 (14.6%)	
≤ 40 U/L	38 (24.5%)	134 (85.4%)	
Genotype, *n* (%)			0.836
Genotype C	132 (85.2%)	135 (86.0%)	0.836
Genotype B	19 (12.3%)	22 (14.0%)	0.646
Genotype other	4 (2.5%)	0 (0%)	0.128

### 3.2 Comparison of Test Sensitivity Between ddPCR and qPCR

**FIGURE 1:**
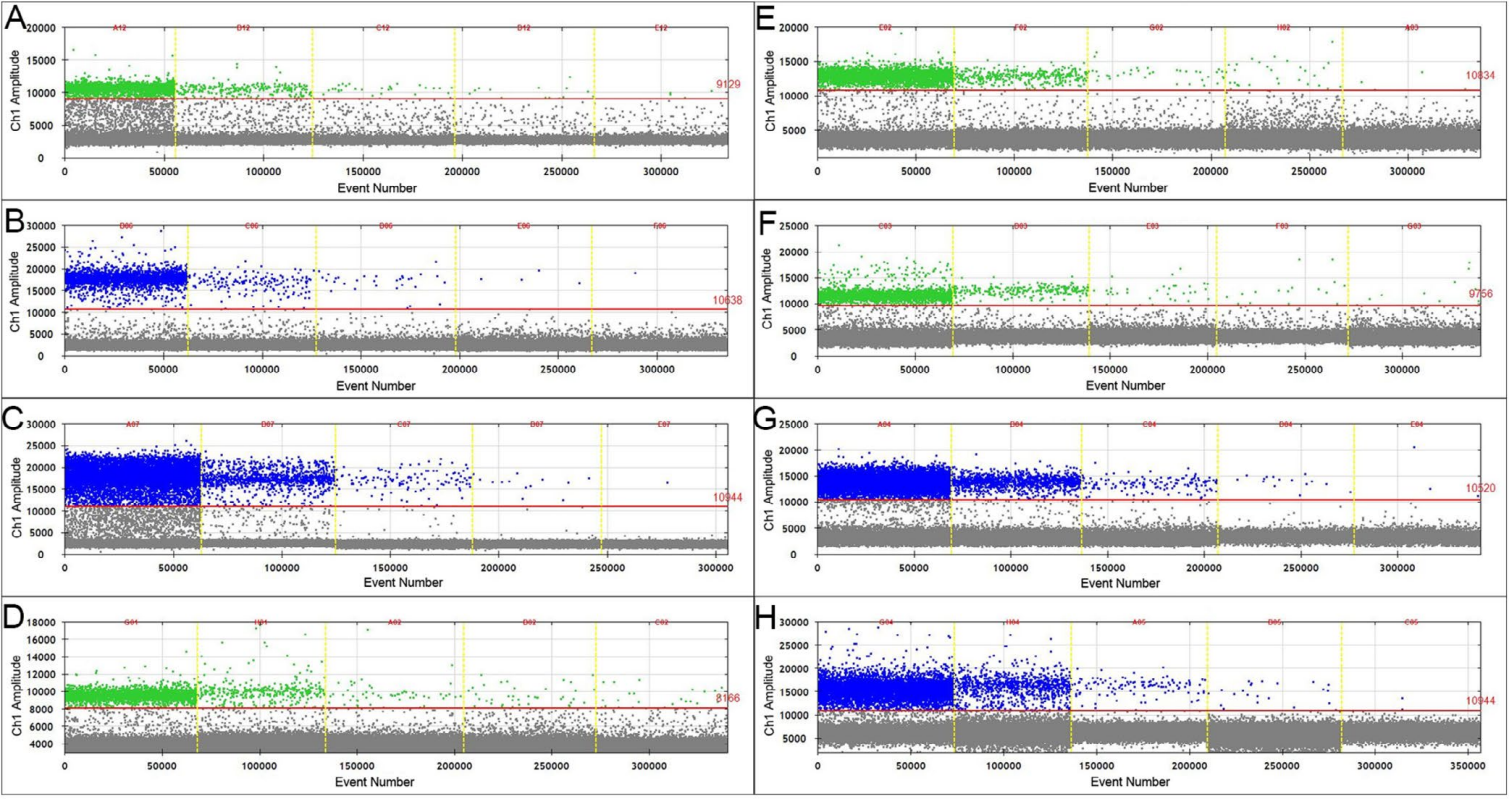
Results of ddPCR experiments for plasmids of wild‐type HBV, G1512A, A1630G, T1753C, T1753G, T1753A, A1762T, and G1764A, labeled (A–H) respectively. The concentration of positive droplets corresponds to dilution factors from10^6^ to 10^8^. Even at a dilution of 10^10^, extremely low concentrations of target sample copies can still be detected.

**FIGURE 2:**
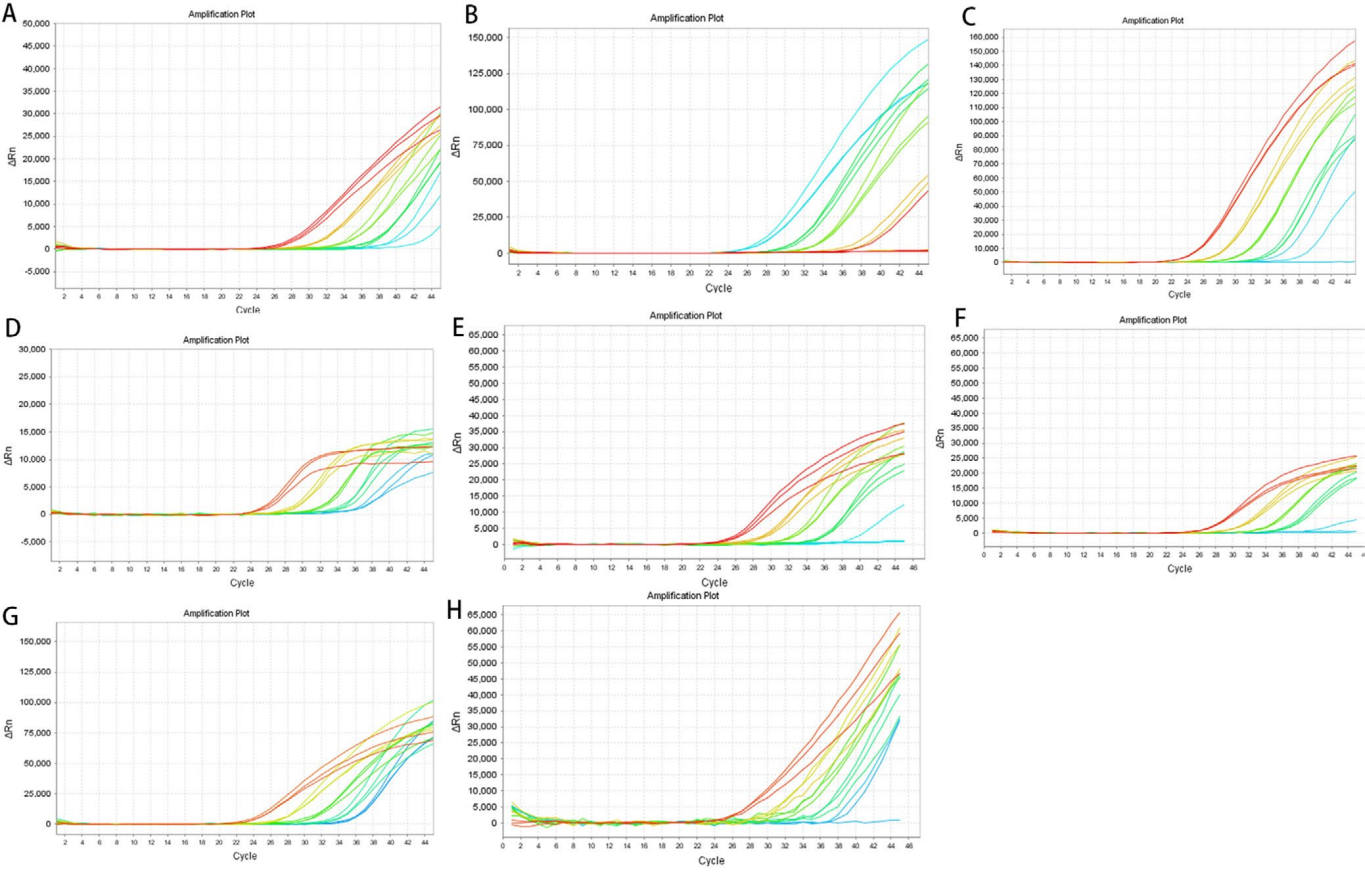
Results of qPCR experiments for plasmids of wild‐type HBV, G1512A, A1630G, T1753C, T1753G, T1753A, A1762T, and G1764A, labeled (A–H) respectively. At a dilution of 10^8^, the cycle threshold values exceed 35, and some samples show no amplification curves, indicating low reliability of the detection results.

The ddPCR and qPCR assays developed in this study demonstrated the capability to accurately detect eight different types of HBV plasmids. As shown in Figure 1 and Table S4, the ddPCR concentration gradient experiment revealed a direct link between the concentration of positive droplets and the dilution factor, ranging from 10^6^ to 10^8^. Remarkably, even at a dilution factor of 10^10^, extremely low concentrations of target sample copies were detectable, indicating exceptionally high sensitivity of the assay. In contrast, the qPCR concentration gradient experiments (Figure 2 and Table S5) revealed that at a dilution factor of 10^8^, the cycle threshold (CT) value exceeded 35, with amplification curves absent in some samples, indicating lower reliability for detecting HBV mutations. A comparative analysis of the two methods confirmed that ddPCR had higher sensitivity compared to qPCR, allowing the detection of HBV mutant plasmids at significantly lower concentrations. These findings underscored the advantage of ddPCR in detecting low‐concentration, low‐copy HBV mutation fragments, particularly in the early detection of HCC from blood samples.

### 3.3 Identification of RFs for HCC

To identify the RFs for HCC, we conducted univariate logistic analysis on 19 variables related to HCC screening, HBV infection status, and HBV variations data. Results showed that G1512A+A1630G multi‐mutations, G1764A mutation, A1762T+G1764A multi‐mutations, T1753C/G/A+A1762T+G1764A multi‐mutations, HBV DNA copy number, HBsAg, and ALT were significantly linked to the incidence of HCC (Table 2). Furthermore, multivariable logistic regression analysis revealed that A1762T+G1764A multi‐mutations (*p* = 0.0395), T1753C/G/A+A1762T+G1764A multi‐mutations (*p* = 0.0372), and ALT > 40 U/L (*p* < 0.0001) were independent predictive RFs for HCC (Table 2). Additionally, we conducted univariate logistic analysis on 9 variables excluding HBV mutation data. The results showed that HBV DNA copy number, HBsAg, and ALT were significantly associated with the incidence of HCC (Table 3). Furthermore, multivariable logistic regression analysis demonstrated that only ALT > 40 U/L (*p* < 0.0001) was an independent predictive factor for HCC (Table 3).

**TABLE 2:** Results of single‐factor and multiple‐factor logistic regression analyses for the training set with HBX mutations data.

Characteristics	Total (*N*)	Univariate analysis		Multivariate analysis	
		Odds ratio (95% CI)	*p*	Odds ratio (95% CI)	*p*
G1512A+A1630G	218				
0	140	Reference		Reference	
1	78	0.483 (0.274–0.849)	**0.012**	0.622 (0.294–1.319)	0.216
G1764A	218				
0	165	Reference		Reference	
1	53	1.932 (1.025–3.643)	**0.042**	0.554 (0.175–1.752)	0.315
A1762T+G1764A	218				
0	161	Reference		Reference	
1	57	0.484 (0.260–0.901)	**0.022**	0.312 (0.103–0.946)	**0.040**
T1753C/G/A+A1762T+G1764A	218				
1	70	Reference		Reference	
0	148	1.813 (1.018–3.231)	**0.043**	3.128 (1.070–9.143)	**0.037**
HBV DNA	218				
0	75	Reference		Reference	
1	143	0.351 (0.196–0.630)	**< 0.001**	0.708 (0.315–1.593)	0.404
HbsAg	218				
1	189	Reference		Reference	
0	29	3.685 (1.502–9.039)	**0.004**	1.825 (0.556–5.992)	0.321
ALT	218				
1	97	Reference		Reference	
0	121	16.815 (8.496–33.278)	**< 0.001**	14.131 (6.968–28.657)	**< 0.001**

*Note:* The bold values presented that the *p* values were less than 0.05 and that was considered statistically significant.

**TABLE 3:** Results of single‐factor and multiple‐factor logistic regression analysis for the training set without HBX mutation data.

Characteristics	Total (*N*)	Univariate analysis		Multivariate analysis	
		Odds ratio (95% CI)	*p*	Odds ratio (95% CI)	*p*
HBVDNA	218				
0	75	Reference		Reference	
1	143	0.351 (0.196–0.630)	**< 0.001**	0.636 (0.297–1.365)	0.246
HbsAg	218				
1	189	Reference		Reference	
0	29	3.685 (1.502–9.039)	**0.004**	2.279 (0.720–7.217)	0.161
ALT	218				
1	97	Reference		Reference	
0	121	16.815 (8.496–33.278)	**< 0.001**	15.172 (7.582–30.362)	**< 0.001**

*Note:* The bold values presented that the *p* values were less than 0.05 and that was considered statistically significant.

### 3.4 Establishment of Two Nomograms for the Risk Assessment of HCC

**FIGURE 3:**
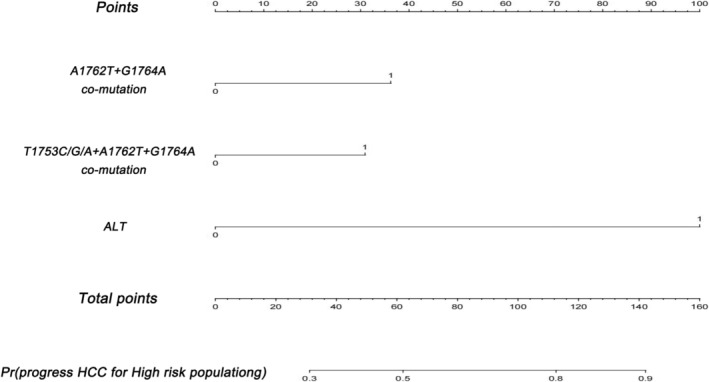
Nomogram with HBX mutation data for predicting HCC in the high‐risk population.

**FIGURE 4:**
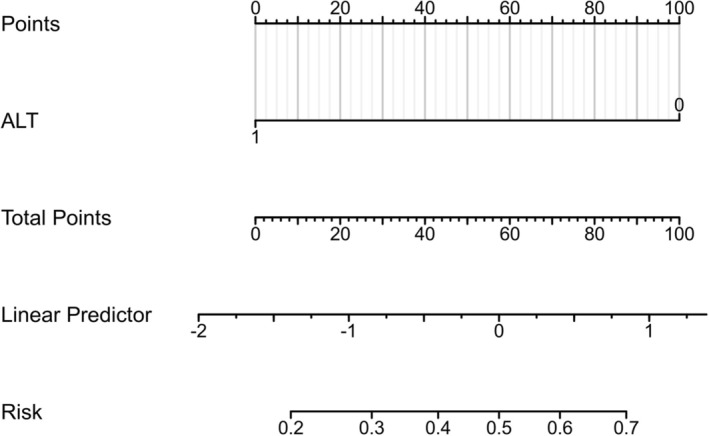
Nomogram without HBX mutation data for predicting HCC in the high‐risk population.

**FIGURE 5:**
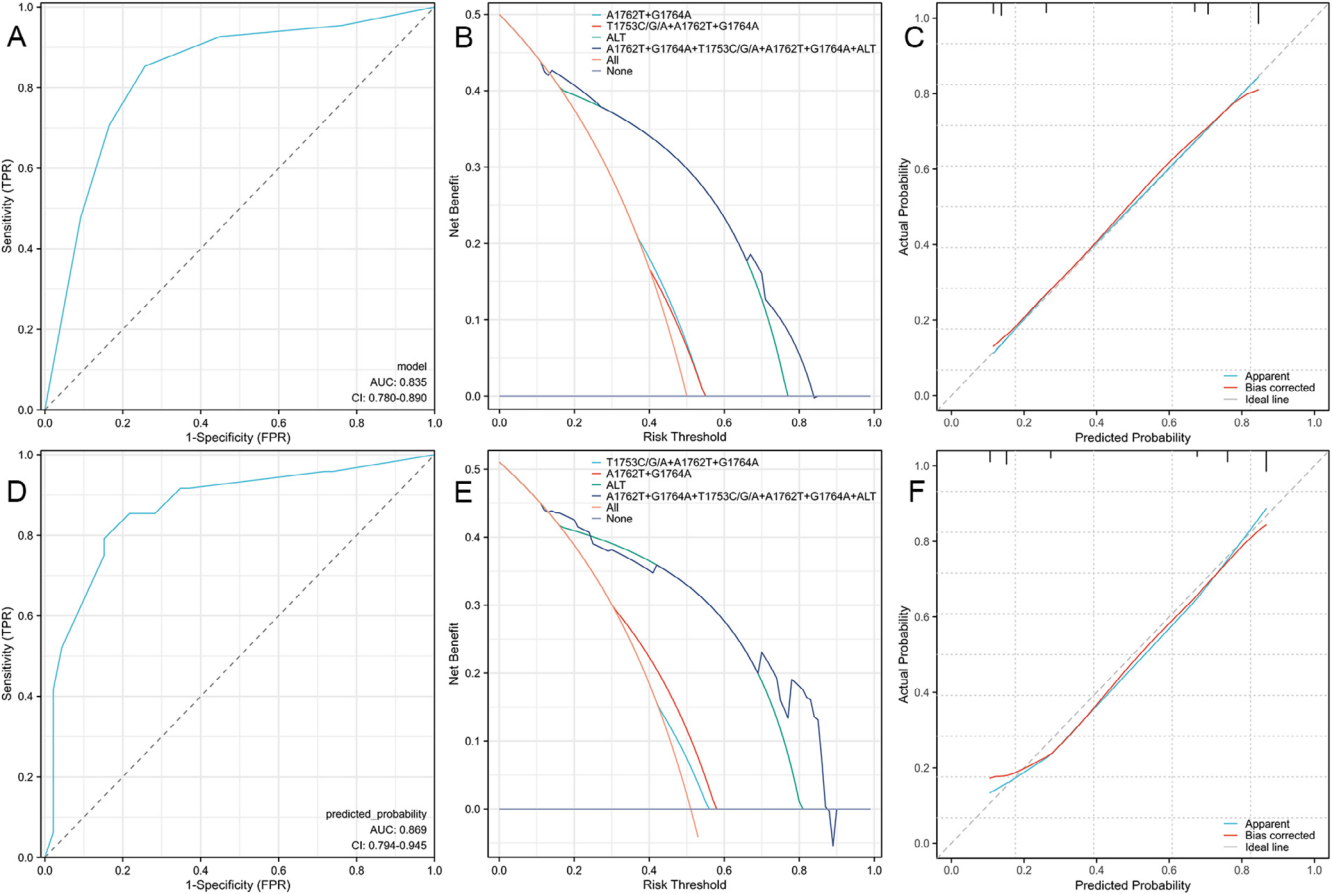
Receiver operating characteristic curve (A), calibration curve (B), and decision curve analysis (C) for the training set of the nomogram with HBX mutation data. The receiver operating characteristic curve (D), calibration curve (E), and decision curve analysis (F) for the testing set of the nomogram with HBX mutation data.

**FIGURE 6:**
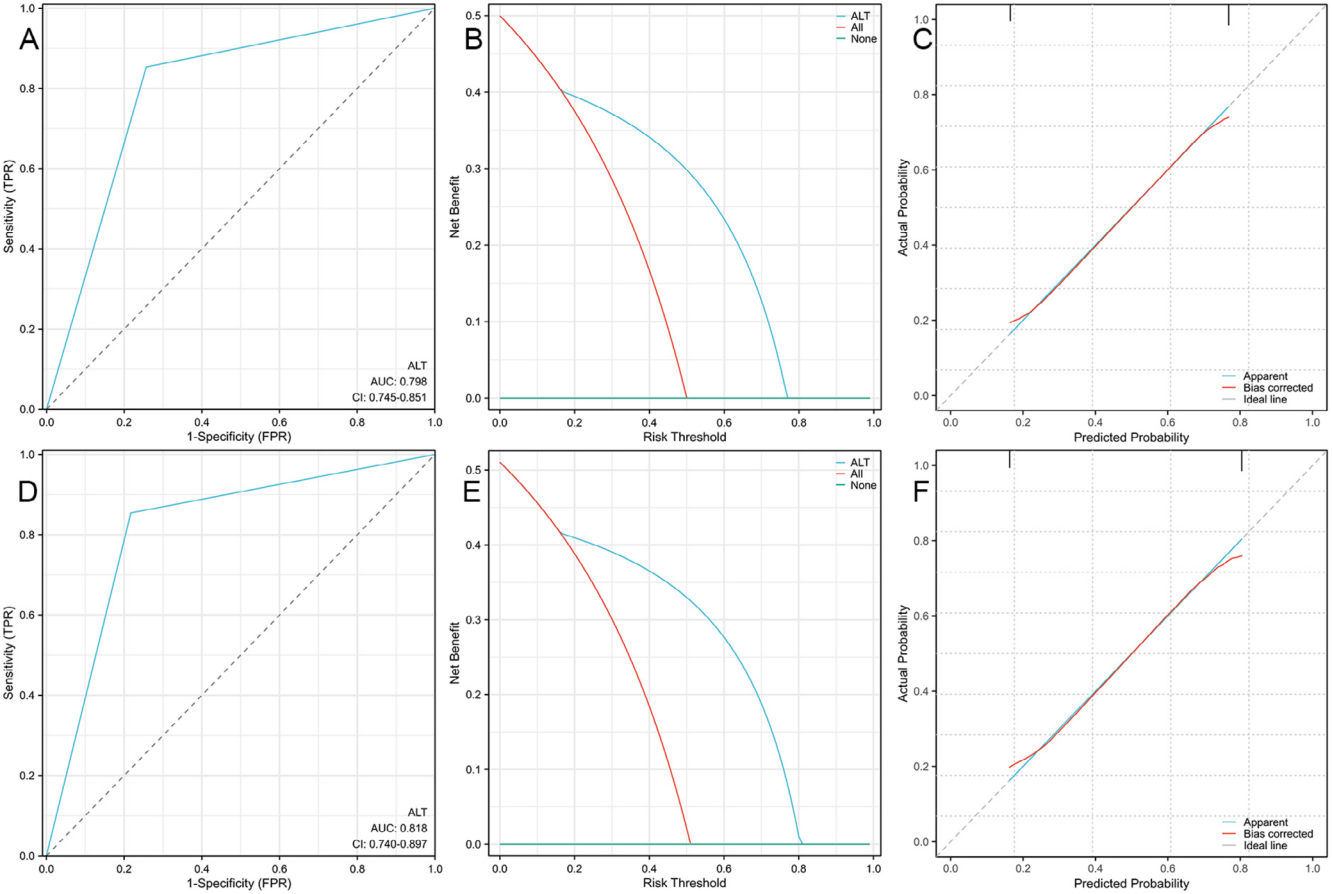
Receiver operating characteristic curve (A), calibration curve (B), and decision curve analysis (C) for the training set of the nomogram without HBX mutation data. The receiver operating characteristic curve (D), calibration curve (E), and decision curve analysis (F) for the testing set of the nomogram without HBX mutation data.

Using the three independent HCC RFs identified above in the dataset with HBX mutations and the single independent RF identified in the dataset without HBX mutations, two nomograms were established to comprehensively assess the risk of HCC and calculate the weight of each RF (Figure 3 and Figure 4). Additionally, ROC curves were drawn for both the training and testing sets. The AUC of the nomogram incorporating HBX mutation data was 0.835 in the training set and 0.869 in the testing set (Figure 5A,D). In contrast, the AUC of the nomogram without HBX mutations was 0.798 in the training set and 0.818 in the testing set (Figure 6A,D). Calibration curves for both training and testing sets demonstrated excellent calibration of the two nomograms (Figure 5C,E). DCA showed that the two nomograms (Figure 5B,E) could serve as effective tools for predicting the risk of HCC (Figure 4). Compared to the nomogram relying solely on traditional risk indicators, the nomogram with HBX mutation indicators had better predictive performance.

## 4 Discussion

In China, over 90% of patients with HCC are associated with HBV infection, which leads to continuous replication and integration within the body, ultimately resulting in HCC development [12]. During replication, the lack of proofreading by DNA polymerase results in a high error rate, contributing to frequent HBV mutations. Various mutations, including A1630G, T1753C, and the region spanning nt1753–1764, have been identified in the HBV X gene and are closely associated with HCC development and progression [13, 14, 15, 16, 17, 18]. Evidence suggests that the presence of double mutations, such as 1762 T/G1764A, and other variant sites can be detected up to 10 years before an HCC diagnosis, particularly in the enhancer II/core promoter sequence, highlighting the significant role of HBV variants in promoting HCC development [19, 20, 21, 22, 23, 24]. In our previous research, we identified seven dominant HBV mutation types during the progression from “HBsAg carrier” to HCC, including G1512A, A1630G, T1753C, T1753G, T1753A, A1762T, as well as G1764A mutations [8]. We believe that monitoring the dynamic changes of these dominant mutations in HR populations is crucial for predicting hepatocarcinogenesis. However, considering the high replication and mutation rates of HBV, together with the lack of feasible detection technology, relevant research has been limited in large‐scale populations.

Traditional PCR technology faces limitations in simultaneously detecting multiple mutations with a single tube. Although NGS offers high precision, its technical complexity and high cost limit its application in mass screening. Therefore, there is a need to prioritize key mutations associated with HCC and explore novel experimental techniques that are suitable for widespread use in HCC screening. Recently, ddPCR has emerged as a promising method for mutation detection, due to its high accuracy and efficiency in identifying low‐copy mutations and capacity to detect multiple mutations within a single reaction [25, 26]. ddPCR has been widely applied across various fields, including the quantification of target DNA, microbial analysis, detection of genome changes such as copy number variations, identification of rare sequences, single‐cell analysis, and detection of gene mutation sites. Based on ddPCR technology, we have developed an advanced multiplex ddPCR method capable of detecting multiple HBV mutations. This method was compared to qPCR in this study to evaluate its performance. The results showed that the detection accuracy of this multiplex ddPCR method was 2–3 orders of magnitude higher than qPCR. This finding highlighted the superior sensitivity of ddPCR in detecting low‐concentration gene mutations, confirming its suitability for identifying HBV mutation fragments with potentially low copy numbers during the early stages of HCC or even prior to its onset. Furthermore, this advanced multiplex ddPCR method could simultaneously detect multiple HBV mutations and combined mutations accurately and conveniently, offering lower time and detection costs, making it more suitable for large‐scale screening of HCC. BO's study also demonstrated that ddPCR could accurately detect mutation sites in samples with lower mutation abundance. Specifically, when detecting epidermal growth factor receptor (EGFR) gene mutations in breast cancer, ddPCR was capable of identifying mutations with an abundance as low as 0.1%, whereas qPCR could only detect mutations at a minimum abundance of 1% in plasmids [26]. Researchers have also used multiplex ddPCR to detect common gene indicators in breast cancer, achieving highly consistent results with immunohistochemistry (IHC) detection by simultaneously detecting HER2, ESR1, PUM1, and PGR genes [27]. It is evident that ddPCR technology for detecting nucleotide mutations is becoming a promising trend in the diagnosis and prognosis of diseases.

When developing a liver cancer risk prediction model, it is essential to first consider the HR factors associated with cancer development. Hepatocarcinogenesis is a complex, multi‐step histological process, with viral factors playing a key role. The transformation of normal liver cells into cancer cells involves genetic and epigenetic changes, influenced by environmental factors, diet, and personal lifestyle. Many researchers have developed HBV‐related prediction models to assess the RFs of HCC in screening populations, including the gamma‐glutamyltransferase, age, and gender‐based HCC (GAG‐HCC) system [28], liver stiffness measurement‐based HCC (LSM‐HCC) model [29], and the American Association for the Study of Liver Diseases HC (AASL‐HCC) scoring model. These models primarily focused on liver function indicators and infection indicators such as HBV serology and HBV DNA replication levels, but they seldom incorporate HBV mutations in large‐scale population cohorts. This implies the challenge of determining individual risks for HCC by integrating all predictive factors. In our study, the advanced multiplex ddPCR method has made it possible to detect HR HBX mutations in large‐scale screenings of HR groups for HCC. By combining dominant HBV mutations detected by ddPCR with traditional indicators, and using univariate and multivariate logistic regression algorithms, we established a nomogram model for predicting HCC incidence in these group. Compared to other common predictive methods, such as machine learning, traditional regression algorithms have the advantage of widespread application in the medical field and strong interpretability. Our results revealed that the predictive model with HBX mutation data outperformed traditional models excluding HBX mutation data, suggesting that integrating dominant HBX mutations can improve risk stratification for HCC in HR populations, thereby enhancing the accuracy of clinical decision‐making and monitoring.

In the present study, three independent RFs identified in the HCC prediction model were ALT > 40 U/L, A1762T+G1764A mutations, and T1753C/G/A+A1762T+G1764A multi‐mutations. ALT, a key marker of liver function, has been linked to an increased risk of HCC in patients with chronic hepatitis B (CHB) who have not received antiviral treatment. A study in Hong Kong found that individuals with persistently elevated ALT levels had a 60‐fold higher risk of developing HCC [30]. Similar findings were reported in the Risk Evaluation of Viral Load Elevation and Associated Liver Disease/Cancer HBV (REVEAL‐HBV) study in Taiwan, where elevated ALT levels were associated with a 5‐fold increase in HCC risk among CHB and HBV carriers [31, 32]. Additionally, Du's study demonstrated that elevated ALT, especially during ALT flare, was a strong predictor of HCC in CHB patients undergoing nucleoside analogue therapy, providing valuable monitoring insights for early detection of HCC. Further analysis of the other two RFs, A1762T+G1764A and T1753C/G/A+A1762T+G1764A multi‐mutations, revealed that HBV nt1753, nt1762, and nt1764 mutations, located in the core promoter (BCP)/pre‐core region of the HBV genome, were among the most common mutation sites. Several studies have demonstrated that specific HBX mutations, such as A1762T+G1764A and T1753C/G/A+A1762T+G1764A, significantly contribute to the development of HCC [33]. For instance, the HBx has been shown to promote hepatocarcinogenesis through various molecular mechanisms. HBx can activate signaling pathways such as Wnt/β‐catenin, TGF‐β, and PI3K/Akt, which are known to drive cell proliferation and tumor progression [34]. In a recent study, it was shown that HBx‐mediated activation of STAT3 is crucial for HCC development in the absence of inflammation, highlighting the direct oncogenic potential of HBx [35]. This emphasized the importance of considering the combined effect of multiple mutations in HBV‐infected individuals, rather than focusing on individual mutations. Similarly, research by Siddiqui Zi et al. has suggested that HBX double mutations like A1762T and G1764A, as well as triple mutations (I127T+K130M+V131L and K130M+V131I+F132Y), were associated with the severity and progression of HBV infection. These combined mutations may trigger cell proliferation by regulating the expression of cell cycle control genes, thus accelerating HCC progression [36]. Additionally, mutations like K130V/M131I may play significant roles in fibrosis and sclerosis by inducing reactive oxygen species (ROS) and mitochondrial depolarization [37]. Veazjalali et al. also found that an increase in these mutations was linked to disease progression and liver cirrhosis [38]. In summary, A1762T/G1764A double mutations are more common in patients with CHB, involving Met substitution at codon 130 as well as Ile substitution at codon 131 in the HBX functional region. These substitutions could increase the affinity of HNF1 for the BCP binding site, enhancing HBV replication [39]. During the development of HCC, numerous HBX integration events occur, where both wild‐type (WT)‐HBx and HBx mutants integrate into the human genome, potentially contributing to the development of HCC through genes such as TERT [40, 41]. PAI1 (also known as SERPINE1) is a proteolytic factor with anti‐fibrinolytic and anti‐plasminogen activator activity, which can promote tumorigenesis through its angiogenic and anti‐apoptotic effects [42]. In the tumor microenvironment, tumor‐associated macrophages stimulated by CXCL12 from cancer‐associated fibroblasts upregulate the expression of PAI1. This upregulation facilitates epithelial‐mesenchymal transformation, promoting malignant behavior in HCC cells [43]. Rui Pu's research indicates that HBx mutants can upregulate PAI1 expression, fostering a liver cancer‐promoting microenvironment, enhancing metabolic reprogramming, and accelerating cell cycle progression, ultimately contributing to the development of liver cancer.

The findings in this study highlighted the significant value of ALT > 40 U/L, A1762T+G1764A multi‐mutations, and T1753C/G/A+A1762T+G1764A multi‐mutations in predicting the occurrence of HCC. The clinical significance of these HBX mutations lies in their potential to serve as biomarkers for early detection and risk stratification of HCC. Given their role in promoting HCC development, targeting these mutations and their downstream pathways may offer therapeutic benefits. For example, inhibiting the activity of HBx or disrupting its interaction with host proteins could potentially reduce the risk of HCC in high‐risk populations. Moreover, targeting the signaling pathways activated by HBx, such as the Wnt/β‐catenin or TGF‐β pathways, has been proposed as a promising strategy for treating HBV‐related HCC. These mutations and their associated mechanisms are thus not only critical for understanding the pathogenesis of HCC but also hold significant promise for the development of targeted therapies [34]. In addition, the nomogram constructed using these three independent RFs provides a simple and practical tool for assessing HCC risk. This model could facilitate the implementation of risk stratification in primary healthcare settings, aiding in the identification of HR HCC groups.

However, there were several limitations in this study. It was a retrospective study, leading to potential patient selection bias. The nomogram has not yet been validated in a prospective cohort, and additional external data are required to confirm its utility. These limitations should be addressed in future research.

## 5 Conclusion

In our study, we successfully constructed a ddPCR method for the detection of high‐frequency HBX mutations, making it suitable for large‐scale screening of HR populations for HCC. The established nomograms effectively predict the risk of HCC in these populations with good accuracy, providing a valuable reference for precise stratification of HR populations and HCC screening.

## Author Contributions

**Chao‐Jun Zhang:** conceptualization (equal), data curation (equal), formal analysis (equal), investigation (equal), methodology (equal), project administration (equal), resources (equal), software (equal), supervision (equal), validation (equal), visualization (equal), writing – original draft (equal), writing – review and editing (equal). **Xiao‐Mei Chen:** conceptualization (equal), data curation (equal), formal analysis (equal), investigation (equal), methodology (equal), resources (equal), software (equal), validation (equal), writing – original draft (equal). **Chang Yan:** data curation (equal), formal analysis (equal), funding acquisition (equal). **Rui‐Bo Lv:** data curation (equal), formal analysis (equal), funding acquisition (equal). **Sanchun An:** data curation (equal), formal analysis (equal), funding acquisition (equal), investigation (equal). **Yun‐Xin Gao:** data curation (equal), formal analysis (equal), funding acquisition (equal). **Tian‐Ren Huang:** data curation (equal), formal analysis (equal), funding acquisition (equal), investigation (equal). **Wei Deng:** conceptualization (equal), data curation (equal), formal analysis (equal), resources (equal), software (equal), supervision (equal), validation (equal), visualization (equal).

## Ethics Statement

We confirm that this manuscript has not been published elsewhere and is not under consideration by another journal. All authors have approved the revised manuscript and agreed to the author list and resubmission to Journal of Inflammation Research. This study has obtained approval from the Ethics Committee of Guangxi Medical University Cancer Hospital (NO. 2021‐KY‐045).

## Conflicts of Interest

Yun‐Xin Gao declared that he is a technical consultant with Guangdong Forevergen Medical Technology Co Ltd. and was responsible for the primers and probe design in this study. The other authors declare no conflicts of interest.

## Supporting information


Data S1.

Table S1.

Table S2.

Table S3.

Table S4.

Table S5.

## Data Availability

The authors confirm that the data supporting the findings of this study are available within the article and its Supporting Information.
