# Supplementary material for: HBX Multi‐Mutations Combined With Traditional Screening Indicators to Establish a Nomogram Contributes to Precisely Stratify the High‐Risk Population of Hepatocellular Carcinoma
Source: Cancer Med. 2025 Mar 5;14(5):e70748. doi: 10.1002/cam4.70748 (PMC11880911; doi:10.1002/cam4.70748)
Supplement: Supplementary file 1 — Data S1. [file CAM4-14-e70748-s002.docx]

Supplementary Materials

**Method of ELISA**

In this study, whole blood samples from 312 patients were directly assayed. The samples were divided into assay wells and control wells, with 20 μL of substrate solution added to each. Five microliters of the sample were added to the assay well, pre-warmed at 37℃, and gently shaken to mix. The reaction was allowed to proceed at 37℃ for 30 minutes, followed by the addition of 20 μL of 2,4-dinitrophenylhydrazine solution. Five microliters of the sample were then added to the control well, gently shaken, and reacted at 37℃ for 20 minutes. Afterward, 200 μL of 0.4 mol/L sodium hydroxide solution was added to both the assay and control wells, gently shaken, and left at room temperature for 15 minutes. The OD values of each well were measured at a wavelength of 505 nm using an ELISA reader. The absolute OD value (assay well OD minus control well OD) was substituted into a standard curve to obtain the corresponding ALT, AST, and AFP activity values. While clinical detection methods for ALT, AST, and AFP include colorimetry and continuous detection, the sensitivity and specificity of the enzyme-linked immunosorbent assay (ELISA) surpass these techniques.
