# Supplementary material for: HBX Multi‐Mutations Combined With Traditional Screening Indicators to Establish a Nomogram Contributes to Precisely Stratify the High‐Risk Population of Hepatocellular Carcinoma
Source: Cancer Med. 2025 Mar 5;14(5):e70748. doi: 10.1002/cam4.70748 (PMC11880911; doi:10.1002/cam4.70748)
Supplement: Supplementary file 2 — Table S1. [file CAM4-14-e70748-s004.docx]

**Table S1: Sequences of eight types of HBV plasmids**

| **Type** | **Sequence** |
| --- | --- |
| HBV-WT 1321-1860nt | cttatcggaaccgacaactcagttgtcctctctcggaaatacacctcctttccatggctgctaggctgtgctgccaactggatcctgcgcgggacgtcctttgtctacgtcccgtcggcgctgaatcccgcggacgacccgtctcggggccgtttgggcctctaccgtccccttcttcatctgccgttccggccgaccacggggcgcacctctctttacgcggtctccccgtctgtgccttctcatctgccggaccgtgtgcacttcgcttcacctctgcacgtagcatggagaccaccgtgaacgcccaccaggtcttgcccaaggtcttacacaagaggactcttggactctcagcaatgtcaacgaccgaccttgaggcatacttcaaagactgtttgtttaaagactgggaggagttgggggaggagattaggttaaaggtctttgtactaggaggctgtaggcataaattggtctgttcaccagcaccatgcaactttttcccctctgcctaatcatctcatgttcatgtcctactgttcaagcctccaagctgtgccttgggtggctttggggc |
| HBV-1512  G>A | cttatcggaaccgacaactcagttgtcctctctcggaaatacacctcctttccatggctgctaggctgtgctgccaactggatcctgcgcgggacgtcctttgtctacgtcccgtcggcgctgaatcccgcggacgacccgtctcggggccgtttgggcctctaccgtccccttcttcatctgccgttccgaccgaccacggggcgcacctctctttacgcggtctccccgtctgtgccttctcatctgccggaccgtgtgcacttcgcttcacctctgcacgtagcatggagaccaccgtgaacgcccaccaggtcttgcccaaggtcttacacaagaggactcttggactctcagcaatgtcaacgaccgaccttgaggcatacttcaaagactgtttgtttaaagactgggaggagttgggggaggagattaggttaaaggtctttgtactaggaggctgtaggcataaattggtctgttcaccagcaccatgcaactttttcccctctgcctaatcatctcatgttcatgtcctactgttcaagcctccaagctgtgccttgggtggctttggggc |
| HBV-1630  A>G | cttatcggaaccgacaactcagttgtcctctctcggaaatacacctcctttccatggctgctaggctgtgctgccaactggatcctgcgcgggacgtcctttgtctacgtcccgtcggcgctgaatcccgcggacgacccgtctcggggccgtttgggcctctaccgtccccttcttcatctgccgttccggccgaccacggggcgcacctctctttacgcggtctccccgtctgtgccttctcatctgccggaccgtgtgcacttcgcttcacctctgcacgtagcatggagaccaccgtgaacgcccgccaggtcttgcccaaggtcttacacaagaggactcttggactctcagcaatgtcaacgaccgaccttgaggcatacttcaaagactgtttgtttaaagactgggaggagttgggggaggagattaggttaaaggtctttgtactaggaggctgtaggcataaattggtctgttcaccagcaccatgcaactttttcccctctgcctaatcatctcatgttcatgtcctactgttcaagcctccaagctgtgccttgggtggctttggggc |
| HBV-1753  T>C | cttatcggaaccgacaactcagttgtcctctctcggaaatacacctcctttccatggctgctaggctgtgctgccaactggatcctgcgcgggacgtcctttgtctacgtcccgtcggcgctgaatcccgcggacgacccgtctcggggccgtttgggcctctaccgtccccttcttcatctgccgttccggccgaccacggggcgcacctctctttacgcggtctccccgtctgtgccttctcatctgccggaccgtgtgcacttcgcttcacctctgcacgtagcatggagaccaccgtgaacgcccaccaggtcttgcccaaggtcttacacaagaggactcttggactctcagcaatgtcaacgaccgaccttgaggcatacttcaaagactgtttgtttaaagactgggaggagttgggggaggagactaggttaaaggtctttgtactaggaggctgtaggcataaattggtctgttcaccagcaccatgcaactttttcccctctgcctaatcatctcatgttcatgtcctactgttcaagcctccaagctgtgccttgggtggctttggggc |
| HBV-1753  T>G | cttatcggaaccgacaactcagttgtcctctctcggaaatacacctcctttccatggctgctaggctgtgctgccaactggatcctgcgcgggacgtcctttgtctacgtcccgtcggcgctgaatcccgcggacgacccgtctcggggccgtttgggcctctaccgtccccttcttcatctgccgttccggccgaccacggggcgcacctctctttacgcggtctccccgtctgtgccttctcatctgccggaccgtgtgcacttcgcttcacctctgcacgtagcatggagaccaccgtgaacgcccaccaggtcttgcccaaggtcttacacaagaggactcttggactctcagcaatgtcaacgaccgaccttgaggcatacttcaaagactgtttgtttaaagactgggaggagttgggggaggagagtaggttaaaggtctttgtactaggaggctgtaggcataaattggtctgttcaccagcaccatgcaactttttcccctctgcctaatcatctcatgttcatgtcctactgttcaagcctccaagctgtgccttgggtggctttggggc |
| HBV-1753  T>A | cttatcggaaccgacaactcagttgtcctctctcggaaatacacctcctttccatggctgctaggctgtgctgccaactggatcctgcgcgggacgtcctttgtctacgtcccgtcggcgctgaatcccgcggacgacccgtctcggggccgtttgggcctctaccgtccccttcttcatctgccgttccggccgaccacggggcgcacctctctttacgcggtctccccgtctgtgccttctcatctgccggaccgtgtgcacttcgcttcacctctgcacgtagcatggagaccaccgtgaacgcccaccaggtcttgcccaaggtcttacacaagaggactcttggactctcagcaatgtcaacgaccgaccttgaggcatacttcaaagactgtttgtttaaagactgggaggagttgggggaggagaataggttaaaggtctttgtactaggaggctgtaggcataaattggtctgttcaccagcaccatgcaactttttcccctctgcctaatcatctcatgttcatgtcctactgttcaagcctccaagctgtgccttgggtggctttggggc |
| HBV-1762  A >T | cttatcggaaccgacaactcagttgtcctctctcggaaatacacctcctttccatggctgctaggctgtgctgccaactggatcctgcgcgggacgtcctttgtctacgtcccgtcggcgctgaatcccgcggacgacccgtctcggggccgtttgggcctctaccgtccccttcttcatctgccgttccggccgaccacggggcgcacctctctttacgcggtctccccgtctgtgccttctcatctgccggaccgtgtgcacttcgcttcacctctgcacgtagcatggagaccaccgtgaacgcccaccaggtcttgcccaaggtcttacacaagaggactcttggactctcagcaatgtcaacgaccgaccttgaggcatacttcaaagactgtttgtttaaagactgggaggagttgggggaggagattaggttaatggtctttgtactaggaggctgtaggcataaattggtctgttcaccagcaccatgcaactttttcccctctgcctaatcatctcatgttcatgtcctactgttcaagcctccaagctgtgccttgggtggctttggggc |
| HBV-1764  G >A | cttatcggaaccgacaactcagttgtcctctctcggaaatacacctcctttccatggctgctaggctgtgctgccaactggatcctgcgcgggacgtcctttgtctacgtcccgtcggcgctgaatcccgcggacgacccgtctcggggccgtttgggcctctaccgtccccttcttcatctgccgttccggccgaccacggggcgcacctctctttacgcggtctccccgtctgtgccttctcatctgccggaccgtgtgcacttcgcttcacctctgcacgtagcatggagaccaccgtgaacgcccaccaggtcttgcccaaggtcttacacaagaggactcttggactctcagcaatgtcaacgaccgaccttgaggcatacttcaaagactgtttgtttaaagactgggaggagttgggggaggagattaggttaaagatctttgtactaggaggctgtaggcataaattggtctgttcaccagcaccatgcaactttttcccctctgcctaatcatctcatgttcatgtcctactgttcaagcctccaagctgtgccttgggtggctttggggc |
