# Supplementary material for: HBX Multi‐Mutations Combined With Traditional Screening Indicators to Establish a Nomogram Contributes to Precisely Stratify the High‐Risk Population of Hepatocellular Carcinoma
Source: Cancer Med. 2025 Mar 5;14(5):e70748. doi: 10.1002/cam4.70748 (PMC11880911; doi:10.1002/cam4.70748)
Supplement: Supplementary file 3 — Table S2. [file CAM4-14-e70748-s005.docx]

**Table S2** Primer and probe sequences used for ddPCR and qPCR

| Mutation types | Primer and probe |
| --- | --- |
| HBX-G1512A | F：GCCTCTACCGTCCCCTTCTTC |
|  | P：GAGAAGGCACAGACGGGGA |
|  | Probe：CGTTCCGACCGACC |
| HBX-A1630G | F：CCGGACCGTGTGCACTTC |
|  | P：TTGACATTGCTGAGAGTCCAAGA |
|  | Probe：AACGCCCGCCAGGTC |
| HBX-T1753C/G/A | F：ATGCCTACAGCCTCCTAGTACAAAG |
|  | P：ACAGACCAATTTATGCCTACAGCC |
|  | Probe：TTAACCTACTCTCCTCCC |
| HBX-A1762T+G1764A | F:ACAGACCAATTTATGCCTACAGCC |
|  | P：ACGACCGACCTTGAGGCATAC |
|  | Probe-A1762T：AGTACAAAGATCATTAACC |
|  | Probe-G1764A：CCTAGTACAAAGATCATTACC |
| HBV-WT | F：CAACTTTTTCCCCTCTGCCTAAT |
|  | R：AAGCCACCCAAGGCACAG |
|  | Probe：CATCTCATGTTCATGTCCT |
