# Supplementary material for: HBX Multi‐Mutations Combined With Traditional Screening Indicators to Establish a Nomogram Contributes to Precisely Stratify the High‐Risk Population of Hepatocellular Carcinoma
Source: Cancer Med. 2025 Mar 5;14(5):e70748. doi: 10.1002/cam4.70748 (PMC11880911; doi:10.1002/cam4.70748)
Supplement: Supplementary file 4 — Table S3. [file CAM4-14-e70748-s001.docx]

Table S3 Reaction conditions for ddPCR and qPCR

| ddPCR reaction conditions | | |  | qPCR reaction conditions | | |
| --- | --- | --- | --- | --- | --- | --- |
| Temperature(°C) | Time (mm:ss) | Cycles |  | Step | Temperature(°C) | Time |
| 50 | 2:00 | 1 |  | 1 | 95 | 5min |
| 95 | 10:00 | 1 |  | 2 | 95 | 5sec |
| 95 | 0:30 | 45 |  | 3 | 60 | 30sec |
| 60 | 1:00 |  |  | Step 1-3 cycle  30 times | - |  |
| 98 | 10:00 | 1 |  | 4 | 72 | 10min |
| 16 | ∞ |  |  |  | 4 | ∞ |
