# Supplementary material for: HBX Multi‐Mutations Combined With Traditional Screening Indicators to Establish a Nomogram Contributes to Precisely Stratify the High‐Risk Population of Hepatocellular Carcinoma
Source: Cancer Med. 2025 Mar 5;14(5):e70748. doi: 10.1002/cam4.70748 (PMC11880911; doi:10.1002/cam4.70748)
Supplement: Supplementary file 5 — Table S4. [file CAM4-14-e70748-s006.docx]

Table S4 Results of concentration gradient experiments on eight types of HBV plasmids using ddPCR

| Type | Dilution factor | Event number | Copy number concentration | Type | Dilution factor | Event number | Copy number concentration |
| --- | --- | --- | --- | --- | --- | --- | --- |
| HBV-  WT | 1×10^6^ | 2015 | 5.14E+03 | HBV-  T1753G | 1×10^6^ | 3416 | 7.05E+03 |
|  | 1×10^7^ | 242 | 4.89E+02 |  | 1×10^7^ | 375 | 7.68E+02 |
|  | 1×10^8^ | 31 | 6.04E+01 |  | 1×10^8^ | 36 | 7.20E+01 |
|  | 1×10^9^ | 13 | 2.58E+01 |  | 1×10^9^ | 29 | 6.73E+01 |
|  | 1×10^10^ | 4 | 8.23E+00 |  | 1×10^10^ | 3 | 5.95E+00 |
| HBV-  G1512A | 1×10^6^ | 2421 | 5.52E+03 | HBV-  T1753A | 1×10^6^ | 2343 | 4.79E+03 |
|  | 1×10^7^ | 168 | 3.61E+02 |  | 1×10^7^ | 243 | 4.85E+02 |
|  | 1×10^8^ | 31 | 6.09E+01 |  | 1×10^8^ | 33 | 7.02E+01 |
|  | 1×10^9^ | 4 | 8.06E+00 |  | 1×10^9^ | 16 | 3.29E+01 |
|  | 1×10^10^ | 1 | 2.02E+00 |  | 1×10^10^ | 12 | 2.45E+01 |
| HBV-  A1630G | 1×10^6^ | 10860 | 2.65E+04 | HBV-  A1762T | 1×10^6^ | 14821 | 3.36E+04 |
|  | 1×10^7^ | 1103 | 2.51E+03 |  | 1×10^7^ | 1201 | 2.50E+03 |
|  | 1×10^8^ | 121 | 2.66E+02 |  | 1×10^8^ | 147 | 2.91E+02 |
|  | 1×10^9^ | 13 | 3.06E+01 |  | 1×10^9^ | 15 | 2.96E+01 |
|  | 1×10^10^ | 1 | 2.38E+00 |  | 1×10^10^ | 4 | 8.48E+00 |
| HBV-  T1753C | 1×10^6^ | 2612 | 5.45E+03 | HBV-  G1764A | 1×10^6^ | 8003 | 1.61E+04 |
|  | 1×10^7^ | 330 | 7.02E+02 |  | 1×10^7^ | 900 | 2.00E+03 |
|  | 1×10^8^ | 51 | 9.98E+01 |  | 1×10^8^ | 113 | 2.15E+02 |
|  | 1×10^9^ | 39 | 8.03E+01 |  | 1×10^9^ | 22 | 4.23E+01 |
|  | 1×10^10^ | 20 | 4.10E+01 |  | 1×10^10^ | 2 | 3.72E+00 |

Note: 1. Take 2ul of sample diluent for microdroplet detection after 20ul system amplification. 2. Digital and qPCR use the same reaction system
