# Supplementary material for: HBX Multi‐Mutations Combined With Traditional Screening Indicators to Establish a Nomogram Contributes to Precisely Stratify the High‐Risk Population of Hepatocellular Carcinoma
Source: Cancer Med. 2025 Mar 5;14(5):e70748. doi: 10.1002/cam4.70748 (PMC11880911; doi:10.1002/cam4.70748)
Supplement: Supplementary file 6 — Table S5. [file CAM4-14-e70748-s003.docx]

TableS5: Results of concentration gradient experiments on eight types of HBV plasmids using qPCR

| Type | Dilution factor | Well | Fluor | Threshold Cycle (Ct) | Ct Mean | Ct Std. Dev |
| --- | --- | --- | --- | --- | --- | --- |
| HBV  WT | 1*10^6^ | E01 | VIC | 27.35 | 27.52 | 0.21 |
|  |  | E02 | VIC | 27.76 |  |  |
|  |  | E03 | VIC | 27.46 |  |  |
|  | 1*10^7^ | E01 | VIC | 31.11 | 30.96 | 0.19 |
|  |  | E02 | VIC | 30.75 |  |  |
|  |  | E03 | VIC | 31.03 |  |  |
|  | 1*10^8^ | E01 | VIC | 33.86 | 34.18 | 0.28 |
|  |  | E02 | VIC | 34.30 |  |  |
|  |  | E03 | VIC | 34.38 |  |  |
|  | 1*10^9^ | E01 | VIC | 37.46 | 37.08 | 0.35 |
|  |  | E02 | VIC | 36.79 |  |  |
|  |  | E03 | VIC | 36.98 |  |  |
|  | 1*10^10^ | E01 | VIC | 39.15 | 40.50 | 1.90 |
|  |  | E02 | VIC | 42.67 |  |  |
|  |  | E03 | VIC | 39.68 |  |  |
| HBV  G1512A | 1*10^6^ | E01 | FAM | 26.21 | 26.54 | 0.29 |
|  |  | E02 | FAM | 26.75 |  |  |
|  |  | E03 | FAM | 26.67 |  |  |
|  | 1*10^7^ | E01 | FAM | 30.06 | 30.23 | 0.24 |
|  |  | E02 | FAM | 30.51 |  |  |
|  |  | E03 | FAM | 30.12 |  |  |
|  | 1*10^8^ | E01 | FAM | 32.71 | 32.78 | 0.09 |
|  |  | E02 | FAM | 32.88 |  |  |
|  |  | E03 | FAM | 32.76 |  |  |
|  | 1*10^9^ | E01 | FAM | Undetermined | 37.02 | 0.92 |
|  |  | E02 | FAM | 36.37 |  |  |
|  |  | E03 | FAM | 37.67 |  |  |
|  | 1*10^10^ | E01 | FAM | Undetermined | 37.98 | #DIV/0! |
|  |  | E02 | FAM | 37.98 |  |  |
|  |  | E03 | FAM | Undetermined |  |  |
| HBV  A1630G | 1*10^6^ | E01 | FAM | 25.00 | 25.07 |  |
|  |  | E02 | FAM | 25.17 |  | 0.09 |
|  |  | E03 | FAM | 25.05 |  |  |
|  | 1*10^7^ | E01 | FAM | 28.44 | 28.35 | 0.12 |
|  |  | E02 | FAM | 28.21 |  |  |
|  |  | E03 | FAM | 28.41 |  |  |
|  | 1*10^8^ | E01 | FAM | 31.37 | 31.36 | 0.12 |
|  |  | E02 | FAM | 31.48 |  |  |
|  |  | E03 | FAM | 31.25 |  |  |
|  | 1*10^9^ | E01 | FAM | 34.99 | 34.82 | 0.33 |
|  |  | E02 | FAM | 35.02 |  |  |
|  |  | E03 | FAM | 34.43 |  |  |
|  | 1*10^10^ | E01 | FAM | 39.08 | 37.66 | 2.01 |
|  |  | E02 | FAM | 36.24 |  |  |
|  |  | E03 | FAM | Undetermined |  |  |
| HBV  T1753C | 1*10^6^ | E01 | VIC | 26.32 | 25.97 | 0.34 |
|  |  | E02 | VIC | 25.96 |  |  |
|  |  | E03 | VIC | 25.64 |  |  |
|  | 1*10^7^ | E01 | VIC | 29.49 | 29.24 | 0.28 |
|  |  | E02 | VIC | 28.93 |  |  |
|  |  | E03 | VIC | 29.29 |  |  |
|  | 1*10^8^ | E01 | VIC | 32.25 | 32.36 | 0.24 |
|  |  | E02 | VIC | 32.19 |  |  |
|  |  | E03 | VIC | 32.64 |  |  |
|  | 1*10^9^ | E01 | VIC | 34.46 | 34.86 | 0.41 |
|  |  | E02 | VIC | 35.28 |  |  |
|  |  | E03 | VIC | 34.83 |  |  |
|  | 1*10^10^ | E01 | VIC | 36.59 | 36.99 | 0.37 |
|  |  | E02 | VIC | 37.04 |  |  |
|  |  | E03 | VIC | 37.32 |  |  |
| HBV  T1753G | 1*10^6^ | E01 | VIC | 26.15 | 26.56 | 0.39 |
|  |  | E02 | VIC | 26.61 |  |  |
|  |  | E03 | VIC | 26.93 |  |  |
|  | 1*10^7^ | E01 | VIC | 29.81 | 29.78 | 0.30 |
|  |  | E02 | VIC | 29.47 |  |  |
|  |  | E03 | VIC | 30.07 |  |  |
|  | 1*10^8^ | E01 | VIC | 32.52 | 32.91 | 0.34 |
|  |  | E02 | VIC | 33.08 |  |  |
|  |  | E03 | VIC | 33.15 |  |  |
|  | 1*10^9^ | E01 | VIC | 36.05 | 36.26 | 0.28 |
|  |  | E02 | VIC | 36.15 |  |  |
|  |  | E03 | VIC | 36.58 |  |  |
|  | 1*10^10^ | E01 | VIC | 39.98 | 39.98 | #DIV/0! |
|  |  | E02 | VIC | Undetermined |  |  |
|  |  | E03 | VIC | Undetermined |  |  |
| HBV  T1753A | 1*10^6^ | E01 | VIC | 27.80 | 27.87 |  |
|  |  | E02 | VIC | 27.80 |  | 0.13 |
|  |  | E03 | VIC | 28.02 |  |  |
|  | 1*10^7^ | E01 | VIC | 31.11 | 31.10 |  |
|  |  | E02 | VIC | 30.97 |  | 0.13 |
|  |  | E03 | VIC | 31.22 |  |  |
|  | 1*10^8^ | E01 | VIC | 34.54 | 34.34 | 0.17 |
|  |  | E02 | VIC | 34.20 |  |  |
|  |  | E03 | VIC | 34.29 |  |  |
|  | 1*10^9^ | E01 | VIC | 37.42 | 37.07 | 0.32 |
|  |  | E02 | VIC | 37.00 |  |  |
|  |  | E03 | VIC | 36.80 |  |  |
|  | 1*10^10^ | E01 | VIC | Undetermined | 41.91 | #DIV/0! |
|  |  | E02 | VIC | 41.91 |  |  |
|  |  | E03 | VIC | Undetermined |  |  |
| HBV  A1762T | 1*10^6^ | E01 | FAM | 25.20 | 25.00 | 0.31 |
|  |  | E02 | FAM | 25.16 |  |  |
|  |  | E03 | FAM | 24.64 |  |  |
|  | 1*10^7^ | E01 | FAM | 28.18 | 28.15 | 0.05 |
|  |  | E02 | FAM | 28.17 |  |  |
|  |  | E03 | FAM | 28.09 |  |  |
|  | 1*10^8^ | E01 | FAM | 31.63 | 31.36 | 0.29 |
|  |  | E02 | FAM | 31.05 |  |  |
|  |  | E03 | FAM | 31.38 |  |  |
|  | 1*10^9^ | E01 | FAM | 34.67 | 34.36 | 0.43 |
|  |  | E02 | FAM | 34.55 |  |  |
|  |  | E03 | FAM | 33.87 |  |  |
|  | 1*10^10^ | E01 | FAM | 36.38 | 36.23 | 0.15 |
|  |  | E02 | FAM | 36.24 |  |  |
|  |  | E03 | FAM | 36.07 |  |  |
| HBV  G1764A | 1*10^6^ | E01 | FAM | 28.16 | 27.82 | 0.29 |
|  |  | E02 | FAM | 27.66 |  |  |
|  |  | E03 | FAM | 27.63 |  |  |
|  | 1*10^7^ | E01 | FAM | 31.44 | 31.02 | 0.37 |
|  |  | E02 | FAM | 30.87 |  |  |
|  |  | E03 | FAM | 30.73 |  |  |
|  | 1*10^8^ | E01 | FAM | 33.69 | 33.66 | 0.41 |
|  |  | E02 | FAM | 34.05 |  |  |
|  |  | E03 | FAM | 33.23 |  |  |
|  | 1*10^9^ | E01 | FAM | 37.35 | 36.79 | 0.49 |
|  |  | E02 | FAM | 36.43 |  |  |
|  |  | E03 | FAM | 36.60 |  |  |
|  | 1*10^10^ | E01 | FAM | 38.24 | 38.85 | 0.87 |
|  |  | E02 | FAM | Undetermined |  |  |
|  |  | E03 | FAM | 39.47 |  |  |
